# Editorial: The Potential Effects and Mechanisms of Traditional Chinese Medicine on Bone Homeostasis and Remodeling

**DOI:** 10.3389/fendo.2022.969729

**Published:** 2022-07-07

**Authors:** Jun Liu

**Affiliations:** ^1^ Bone and Joint Research Team of Degeneration and Injury, Guangdong Provincial Academy of Chinese Medical Sciences, Guangzhou, China; ^2^ Guangdong Second Traditional Chinese Medicine Hospital (Guangdong Province Enginering Technology Research Institute of Traditional Chinese Medicine), Guangzhou, China

**Keywords:** Chinese traditional medicine, Bone homeostasis, Bone Remodeling, effectiveness, mechanisms

Traditional Chinese medicine (TCM) has a long history in the treatment of clinical diseases, but research efforts are needed to understand its mechanisms. Osteoporosis (OP) and its complications, such as osteoporotic fractures and pain, significantly reduce the quality of life and increase the economic burden for patients (1). The intervention measures commonly used in the treatment of OP include exercise therapy, calcium, vitamins, bisphosphates, etc., but these treatments have limitations or contraindications (2). Clinical applications of additional therapies (including drug therapy) are conducive to providing more choices for the treatment of OP. Although TCM, especially Chinese herbal medicine, has potential efficacy in the treatment of OP (3), it is necessary and urgent to clarify its mechanisms. Therefore, this topic presents the current research results focusing on the mechanisms of TCM in bone homeostasis balance and bone reconstruction to further reveal the specific mechanisms of TCM. This topic has been widely studied in the field of TCM and has led to excellent achievements in clinical and basic research. Based on the collected research results, this editorial further summarizes the role of TCM in bone homeostasis and remodeling.

## Clinical Evidence-Based Results

Quantitative systematic evaluation is an objective method for clinical workers to evaluate the effects of different therapies. Xianling Gubao capsule (XLGB) is often used to treat OP and is considered to have good clinical efficacy. Luo et al. evaluated the effect of XLGB and its combination therapy on bone mineral density (BMD) in a postmenopausal osteoporosis population by network meta-analysis. Their results showed that the combined application of XLGB effectively improved BMD in patients with postmenopausal osteoporosis compared with oral calcium, vitamin D or bisphosphates alone. The results of a study by Cheng et al. suggested that the oral administration of XLGB alone or combined with other conventional anti-osteoporosis drugs improved patients’ quality of life and reduced pain. Although the above two studies provide objective data for the clinical application of XLGB, future clinical research needs stricter design criteria regarding specific details such as the drug dose and course of treatment. The source papers included in Luo and Cheng’s study are all Chinese literature, which may produce evidence bias and reduce the credibility of the evidence. Furthermore, the above two studies used some inappropriate outcome indicators to evaluate the anti-osteoporosis efficacy of XLGB, such as pain, ALP and BGP levels, which should be avoided in future research protocols, and appropriate and standard evaluation indicators should be selected.

## Herbal Monomers

Exploring the efficacy of Chinese herbal medicine monomers in the treatment of disease models through animal or cell experiments is conducive to further promoting the development and utilization of drugs. This topic includes the results of three research teams on the interventions of *luteolin*, *quercetin* and *icariin* in the model of OP. Liang et al. found that *luteolin* reduced bone loss in ovariectomized rats, and further mechanistic studies have suggested that *luteolin* regulates the activity of the PI3K-Akt signaling pathway and promotes osteogenic differentiation of bone marrow stromal cells (BMSCs). Sun et al. discussed the effect and mechanism of *quercetin* on bone mass in mice with OP induced by androgen deprivation therapy. Their results showed that *quercetin* increased bone mass, enhanced bone strength and improved bone microstructure in mice, and its mechanism is regulating glucose and lipid metabolism through the GPRC6A/AMPK/mTOR signaling pathway. The mechanism of *icariin* in the treatment of OP is a hot spot in TCM research. Wang et al. evaluated the effect of *icariin* on the bone mass of ovariectomized rats by observing intestinal flora and metabolites. It was suggested that *icariin* can improve the bone mass of ovariectomized rats, and the results of a metabolomic analysis showed that amino acid and fatty acid metabolism play important roles. These studies explored the role of herbal monomers in the maintenance of bone homeostasis from the perspectives of glucose and lipid metabolism, intestinal flora and metabolomics, giving us a new perspective. In addition, these studies reflect the great potential of herbal monomers in the treatment of OP.

## Single Herbal Medicine

With the development of TCM, a large number of Chinese herbal medicines are used in clinical practice. A single herbal medicine contains multiple compounds or monomers, which creates an extensive resource bank for new drug development. Therefore, it is an important research direction to study the efficacy of a single herbal medicine in the treatment of OP. Zhao et al. used network pharmacology and molecular docking technology to find that there are 12 active components that may become drugs in Polygonati Rhizoma, and these 12 components can directly act on 84 targets related to OP. It has been concluded that Polygonati Rhizoma plays an anti-osteoporosis role by regulating the target genes JUN, TP53, AKT1, ESR1 and CASP3. However, Zhao et al’s discovery is only a preliminary theoretical derivation, and further experimental validation is required to support the reliability of the prediction results. Natural terpenoids commonly exist in Chinese herbal medicine and have been widely studied because they play a key role in physiological and pathological processes such as osteogenesis and bone resorption. Zhou et al. reviewed the source, molecular structure, and research progress of known terpenoids of Chinese herbal medicine and the mechanism of these compounds in the treatment of OP. To the best of my knowledge, this is the most comprehensive review on this topic, which provides a usable basis and clear direction for basic experiments and clinical drug development.

## Traditional Chinese Medicine Compounds

Traditional Chinese medicine compounds are combinations of two or more Chinese herbal medicines according to the theory of TCM and the principle of prescription, which plays an important role in the field of TCM. It is undeniable that due to the complex composition of traditional Chinese medicine compounds, research on the mechanism of TCM treatment has always been difficult. Zhang et al. screened the key targets and pathways of Zhuanggu Busui Formula (ZGBSF) through network pharmacology technology and verified them through strictly designed animal experiments. Their study showed that ZGBSF promotes osteogenesis by inhibiting osteoblast apoptosis and activating the PI3K-Akt signaling pathway, thereby reducing bone loss. Huang et al. showed that modified Duhuo Jisheng Decoction prevents bone loss by inhibiting the inflammatory response and downregulating the expression levels of TNF-α, IL-6 and CASP3. Proteomics is also a promising direction in the study of OP. Through quantitative proteomics, Wei et al. confirmed that Bugu Shengsui Decoction plays an anti-osteoporosis role by regulating the PI3K-Akt signaling pathway. Shen et al. investigated the effects of Zuo-Gui-Wan Aqueous Extract on glucocorticoid-induced rat vertebral bone microstructure, autophagy corpuscles, let-7f and autophagy-related genes through *in vivo* and *in vitro* experiments and concluded that Zuo-Gui-Wan can promote the osteogenic differentiation of BMSCs by activating let-7f and inhibiting autophagy. *Yushen Hezhi* is another important direction in the treatment of OP. Traditional Chinese medicine compounds for the treatment of OP, such as *Yushen Hezhi Formula*, should be studied further. Traditional Chinese medicine compounds are an indispensable part of the theoretical system of TCM, which is considered to play a multicomponent and multitarget therapeutic role and regulate the biochemical network of the body as a whole. The above four studies have examined the treatment of OP with TCM from the perspectives of network pharmacology, quantitative proteomics, autophagy and metabolomics. Due to the complex composition of traditional Chinese medicine compounds, methodological exploration and improvement, multidisciplinary cooperation and the application of new technology in traditional Chinese medicine compound research in the future are key.

## Conclusion

The 11 research results in this special issue are representative of the depth of content and the application of methodology, which has attracted the increasing attention of researchers and confirmed the great potential of TCM in the treatment of OP. Although the content of this topic is limited, these research results also give us great encouragement and confidence. Due to the particularity of TCM, in addition to learning from the existing research technologies and methods, it remains necessary to innovate the research methodology, technology and theory of TCM in the treatment of OP in the future.

## Author Contributions

The author confirms being the sole contributor of this work and has approved it for publication.

## Funding

This research was funded by grants from National key research and development program (2021YFC1712804), the Science and Technology Research Project of Guangdong Provincial Hospital of Chinese Medicine (No.YN2019ML08), Research Fund for Bajian Talents of Guangdong Provincial Hospital of Chinese Medicine (No.BJ2022KY01) andProject of Philosophy and Social Science Planning of Guangzhou in 2022 (No.2022GZQN42).

## Conflict of Interest

The author declares that the research was conducted in the absence of any commercial or financial relationships that could be construed as a potential conflict of interest.

## Publisher’s Note

All claims expressed in this article are solely those of the authors and do not necessarily represent those of their affiliated organizations, or those of the publisher, the editors and the reviewers. Any product that may be evaluated in this article, or claim that may be made by its manufacturer, is not guaranteed or endorsed by the publisher.
